# Editorial: COVID-19 and Existential Positive Psychology (PP2.0): The New Science of Self-Transcendence

**DOI:** 10.3389/fpsyg.2021.800308

**Published:** 2021-12-08

**Authors:** Paul T. P. Wong, Claude-Hélène Mayer, Gökmen Arslan

**Affiliations:** ^1^Department of Psychology, Trent University, Peterborough, ON, Canada; ^2^Department of Industrial Psychology and People Management, University of Johannesburg, Johannesburg, South Africa; ^3^Department of Psychology, Mehmet Akif Ersoy University, Burdur, Turkey; ^4^The University of Melbourne, Melbourne, VIC, Australia

**Keywords:** transcendence, suffering, flourishing, COVID-19, existential positive psychology (PP2.0)

COVID-19 has changed everything. It has brought devastations and disruptions (Gallup, 2020; Harvard University, 2021), but in the midst of devastations there is transformation and innovation. This special issue of Frontiers provides an introduction to the new science of flourishing through suffering (Wong, 2021a) and a new vista of meaning-centered global wellbeing (Batthyany and Russo-Netzer, 2014; Wong, 2017a; DeAngelis, 2018). The transformative power of suffering can be seen at various levels.

At the individual level, the adversity and lockdown from COVID-19 provide opportunities for business growth (Valinsky, 2020; Peek and Casarella, 2021) and personal transformation (Weir, 2020; Williams et al., 2021; Kim et al.). In short, it is possible that the worst of times can become the best of times for any individual with the necessary inner resources such as meaning, faith, courage, and creativity (Wong and Worth, 2017; Marano, 2021).

At the institutional level, the ever-evolving coronavirus has laid bare the inadequacies of the current medical model and the need for change in government policies in funding and mental healthcare (Wong, 2021b). For example, we may need more grass-roots mental health education (International Network on Personal Meaning, 2019) and more accessibility to different kinds of qualified mental health providers which are currently not supported by governments and insurance companies. We may also need to consider a more humanistic-existential orientation toward mental health as advocated by Rollo May (Schlett, 2021) and Victor Frankl (Wong, 2021c,d).

At the cultural level, the pandemic represents a wake-up call to the weaknesses of an individualist and pleasure-seeking society like America in combating the coronavirus (Friedman, 2020). More specially, the anti-vaccination movement in the name of personal freedom and liberty reveals the fault-line in democracy: freedom without personal and social responsibility may endanger public health in terms of rising COVID cases (Gerson, 2021) and new waves of the pandemic (Aiello, 2021). The freedom to pursue happiness and pleasure without responsibility may be killing Western democracy because democracy without responsibility may degenerate to nihilism or succumb to authoritarianism.

## What is Existential Positive Psychology? Why is it Necessary?

### Times and Circumstances Have Changed

Seligman (1999) launched his brand of positive psychology at a time of peace and prosperity, but much has changed since 9/11. Things just got even worse with the pandemic. The first two decades of the 21st century seem to be more challenging for humankind than the Bill Clinton years that gave birth of PP1.0.

With COVID-19, the universality and importance of human suffering can no longer be denied or ignored even by the die-hard optimists. There is irrefutable evidence that the pandemic has caused immense human suffering globally (CAMH, 2020; Hwang et al., 2020; Andrian and Ming, 2021; Khan et al., 2021). The death tolls and new cases are constantly on the mass media. Moreover, the need for vaccination, masking, and social distancing reminds us daily of the real and present danger of the coronavirus and its variants. COVID-19 poses as an existential crisis for both the young and old.

### Existential Suffering

Most human suffering can be classified as existential suffering. “Themes common to the descriptions of existential suffering included lack of meaning or purpose, loss of connectedness to others, thoughts about the dying process, struggles around the state of being, difficulty in finding a sense of self, loss of hope, loss of autonomy, and loss of temporality.” This type of suffering calls for different ways of coping and interventions. Bates (2016) suggested that physicians can be trained to address existential suffering so that “it is possible for patients to transition from feeling hopeless to feeling more alive than ever.” More recently, Wong and Yu (2021) point out that even the dreaded time of dying can be transformed to a time of deep joy and liberation from human bondage through radical acceptance, faith in God, and support from family and community. That is why the mission of existential positive psychology is to investigate ways to reduce human suffering and transform it into human flourishing (Wong, 2009a, 2011, 2019a, 2021a).

In sum, just as the nature of mental illness and psychotherapy changed a great deal right after the Second World War (Schlett, 2021), so does the nature of mental health needs in the era of COVID-19. The happy song of positive psychology may sound jarring for those who are struggling and suffering. Sad songs (e.g., Honey and Wong, 2021) may be more comforting to the suffering people (Nield, 2016; Paulus, n.d.). Indeed, there are different kinds of wellbeing and happiness for different seasons of life (Lee, 2021).

Existential positive psychology represents a unique kind of second wave positive psychology because it embraces the human complexity of existentialism and Taoism. PP2.0 goes beyond mere recognition of polarity, and it makes the bold assumptions that (a) suffering is necessary for flourishing and (b) that enduring happiness and wellbeing can only be achieved through the dialectical integration of opposites. The metaphor of a piano keyboard can best illustrate the unique nature of PP2.0: beautiful music can only be produced by engaging both the black and white keys. As a caveat, in this special issue, EPP and PP2.0 are used interchangeably, but keep in mind that here, PP2.0 represents the synthesis between thesis (existentialism) and antithesis (positive psychology).

## The Empirical Basis for the New Science of Flourishing Through Suffering

EPP (PP2.0), as explained above, is probably the most exciting development in positive psychology and mental health, not only because it changes the paradigm of wellbeing research and deepens our understanding of suffering, but also because it provides a rich arsenal of meaning-centered diagnostic and intervention tools (Wong, 2019b, 2021b; Arslan and Wong, 2022; Wong et al.). The following selective review highlights empirical support of for the transformative power of suffering.

### Suffering is Redemptive

Suffering can restore our soul by making us aware of our need to be connected with our spiritual dimension, or spiritual values such as compassion, humility, forgiveness, connections, and self-transcendence. This theme has a venerable history in psychology from James' (1902/1912) need of rebirth for the sick soul, Jung (1949)'s search for the hidden soul, Frankl's (1985) unconscious God, and McAdams's (2013) redemptive self.

Therefore, global wellbeing may depend on meeting our deep-seated yearnings for love, meaning, and faith. It seems reasonable to conclude that faith in God or a higher power, loving relationships, and meaningful work are essential for mental health (Mayer, 2017, 2021a,b; Mayer and Viviers, 2018; Mayer and Fouché, 2021) just as food, water, and air are essential for our physical health.

Self-transcendence remains one the main themes in the new science of suffering (Frankl, 1946/1985; Wong, 2016; Kaufman, 2020) because it has the dual function of transcending personal limitations and the dark side of human existence and connecting with God and with humanity. It does not matter whether one lives a privileged and luxurious life, or in a life of conditions of poverty or traumatic stress; we all have the capability to react to adversity with self-transcendence or with anger and despair. We have the responsibility to choose to become better or bitter in a traumatic situation; that is why not all people experience post-traumatic growth.

In the present issue, Wong et al. supports the thesis that suffering triggers the search for meaning, or self-transcendence, which in turn function as a buffer against the adverse effects of suffering. This issue features other studies on self-transcendence (Braun-Lewensohn et al.; Flotman; Kim et al.; Liu et al.; Mayer and May; Russo-Netzer and Ameli; Worth and Smith).

### Suffering Can Increase Our Resilience

Suffering can increase our resilience by deepening our character strengths and broadening our capacity to cope. There is a vast literature on this topic because as an umbrella concept, suffering encompasses a number of related areas, such as pain, disease, stress, trauma, unpleasant emotions, hardships, conflicts, inner demons, and fear of death. For example,

Bueno-Gómez's (2017) conceptualization of suffering includes many unpleasant aspects of life:

“Pain can be a source of suffering, but it is not the only one. Social problems like poverty, social exclusion, forceful social inclusion (like peer pressure), forced displacement and uprooting; existential and personal problems like grief and stress; conditions like nausea, paresthesia, a non-painful illness, anxiety or fear can likewise be a cause of suffering.”

Thus, by including the existential dimension we can transform suffering into flourishing, as we argue in this editorial. There is considerable empirical support for this positive view of suffering (Arslan and Yildirim; Cox et al.; González-Tovar and Hernández-Rodríguez; Israelashvili; Parrott et al.; Reed et al.; van Zyl et al.; Waters et al.; Wilkie et al.; Yildirim and Arslan; Zhao et al.). For example, Liu (2015) observed that “In what has been termed the ‘steeling effect,' ‘stress inoculation,' and ‘antifragility,' exposure to moderate stressors early in life may confer resilience to potential detrimental effects of later stressors.”

Likewise, Wong has published extensively how proactive and transformative coping with stress and suffering can increases one's resilience and wellbeing (Wong, 1989, 1993, 1995; Wong et al., 2006a,b; Wong and Tomer, 2011). Recent research on overcoming the triad of ancient dark emotions—guilt, shame, and fear (Wong, 2019c; Mayer et al., 2021; Langewitz; Mayer and Vanderheiden) and the tragic triad of guilt, suffering, and death (Lukas, 1990) provides additional support for our thesis.

The present special issue provides more empirical support for the thesis that the most promising approach to cope with suffering is through (a) meaning-focused coping (Arslan and Yildirim; Arslan et al.; Ashraf et al.; Bergman et al.; Eisenbeck et al.; Enea et al.; Karataş et al.; Mayer; Quiroga-Garza et al.; Sanchez-Ruiz et al.); and (b) self-transcendence (Braun-Lewensohn et al.; Flotman; Kim et al.; Liu et al.; Mayer and May; Russo-Netzer and Ameli; Worth and Smith). The importance of existential courage is also an essential aspect of resilience (Chen et al.; Fowers et al.; Leung et al.).

In sum, resilience is no longer defined only in terms of bouncing back. Rather, PP2.0 posits that the development of a resilient mindset (Wong, 2020a,b) and the cultivating of inner resources (Wong et al., 2006a; Wong, 2017b) represent a proactive way of coping with trauma, leading to true grit and resilience. This new approach involves a more realistic and existential way of viewing the world as full of suffering, but also full of overcoming (Apter, 2020; Vozza, 2020). All the good things we value and cherish are on the other side of suffering; we will not be able to fulfill our dreams without a resilient mindset to embrace sacrifices and to go through the gates of fears and suffering (Wong, 2020b).

### Suffering Provides New Grounds for Hope and Happiness

Suffering teaches us life intelligence and calm-based mature happiness as an antidote to a shallow view of life. Wong's has published extensively on tragic optimism and mature happiness (Wong and McDonald, 2002; Wong, 2009b, 2017c; Wong and Bowers, 2018). An existential perspective enables us to see life as it is and yet with a trauma-informed positivity.

Sustainable wellbeing can be achieved through learning how to make the best use of the dynamic and dialectic interplay between positive and negative life experiences in each context. The ancient Yin-Yang dialectic or the contemporary dual-system model (Wong, 2012) provides a blueprint of how to navigate between opposite forces, such as good and evil, and self and other, which are prevalent in life (Lomas and Ivtzan, 2016; Wong and Bowers, 2018; Deng et al., 2020; Wong, 2020c).

To succeed in life or achieve wellbeing, one needs to find the right balance between Yin and Yang. In other words, PP2.0 represents the complete circle or the wholeness of wellbeing in which Yin and Yang co-exist in optimal balance and harmony as shown in the Yin-Yang symbol.

Several papers in this issue show that suffering can lead to deeper joy, inner harmony, or calm-based happiness (Carreno et al.; Chen et al.; Robbins; Wasowicz et al.); existential gratitude and altruistic behavior (Al-Refae et al.; Jans-Beken; Kotera and van Gordon; Kotera et al.); and a sense of tragic optimism (Leung et al.; Mead et al.) through dialectics and courage (Bai et al.; Ferreira et al.; Rajkumar; Van Tongeren and Showalter Van Tongeren).

## Conclusion

The collection of articles in this special issue represents a rich tapestry of PP2.0 and new vistas for global wellbeing research. We are glad that a forthcoming textbook on positive psychology is based on the existential perspective (Wong, 2021e; Worth, 2021). We believe that additional books and articles will follow.

The take-home message of this special issue is that in the final analysis, all our efforts to advance global wellbeing and human flourishing will not succeed until we recognize and address the different sources of suffering. This conclusion is no different from medical science– physical health and public health cannot be achieved without controlling diseases and pathogens. Nature could be cruel. Life could be crueler. It is hard to understand how anyone can survive and thrive a pain-ridden world without faith, hope, and love; these ideals can only be achieved by sinking our roots into deepest Hell and reaching toward the highest Heaven. This special issue of Frontiers draws attention to the missing link in wellbeing research: the existential positive psychology of transcending and transforming inescapable suffering into flourishing.

## Author Contributions

PW wrote the first draft of the manuscript. All authors contributed to manuscript revision, read, and approved the submitted version.

## Conflict of Interest

The authors declare that the research was conducted in the absence of any commercial or financial relationships that could be construed as a potential conflict of interest.

## Publisher's Note

All claims expressed in this article are solely those of the authors and do not necessarily represent those of their affiliated organizations, or those of the publisher, the editors and the reviewers. Any product that may be evaluated in this article, or claim that may be made by its manufacturer, is not guaranteed or endorsed by the publisher.

## References

[B1] Aiello R. (2021). Canada is heading towards a ‘Delta-driven' fourth wave, Tam says. CTV News. Available online at: https://www.ctvnews.ca/health/coronavirus/canada-is-heading-towards-a-delta-driven-fourth-wave-tam-says-1.5529290 (accessed October 1, 2021).

[B2] Andrian T. Ming D. (2021). Human Suffering During Pandemic COVID-19. Available online at: https://www.researchgate.net/publication/348350135_HUMAN_SUFFERING_DURING_PANDEMIC_COVID_19 (accessed October 1, 2021).

[B3] Apter T. (2020). Anxiety Management and the Paradox of Trigger Warnings. Psychology Today. Available online at: https://www.psychologytoday.com/ca/blog/domestic-intelligence/202009/anxiety-management-and-the-paradox-trigger-warnings (accessed October 1, 2021).

[B4] Arslan G. Wong P. T. P. (2022). Measuring personal and social responsibility: an existential positive psychology approach. J Happiness Health 2, 1–11. 10.47602/johah.v2i1.5

[B5] Bates A. T. (2016). Addressing existential suffering. BCMJ 58, 268–273. Available online at: https://bcmj.org/articles/addressing-existential-suffering

[B6] Batthyany A. Russo-Netzer P. (eds.). (2014). Meaning in Positive and Existential Psychology. New York, NY: Springer.

[B7] Bueno-Gómez N. (2017). Conceptualizing suffering and pain. Philos Ethics Humanit Med. 12:7. 10.1186/s13010-017-0049-5 28958214 PMC5621131

[B8] CAMH. (2020). Mental Health and the COVID-19 Pandemic. Available online at: https://www.camh.ca/en/health-info/mental-health-and-covid-19 (accessed October 1, 2021).

[B9] DeAngelis T. (2018). In search of meaning. Monit Psychol. 49, 38–44. Available online at: https://www.apa.org/monitor/2018/10/cover-search-meaning

[B10] Deng K. Wong Y. J. Li J. P. F. McCullough K. M. (2020). Dialectical coping and well-being among Chinese college students: the mediating role of resilience. Counsell. Psychol. Q. 10.1080/09515070.2020.1783641

[B11] Frankl V. E. (1946/1985). Man's Search for Meaning. New York, NY: Washington Square Press.

[B12] Frankl V. E. (1985). The Unconscious God. New York, NY: Washington Square Press.

[B13] Friedman U. (2020). Why America is Uniquely Unsuited to Dealing With the Coronavirus. The Atlantic. Available online at: https://www.theatlantic.com/politics/archive/2020/03/coronavirus-united-states-vulnerable-pandemic/608686/ (accessed October 1, 2021).

[B14] Gallup. (2020). How Leaders Are Responding to COVID-19 Workplace Disruption. Available online at: https://www.gallup.com/workplace/307622/leaders-responding-covid-workplace-disruption.aspx (accessed October 1, 2021).

[B15] Gerson M. (2021). Opinion: Republicans who support anti-vaccine bills are engaging in performative libertarianism. *The Washington Post*. Available online at: https://www.washingtonpost.com/opinions/2021/07/26/sununu-medical-freedom-vaccine-bills-endanger-public-health/ (accessed October 1, 2021).

[B16] Harvard University. (2021). Office of the Dean: The Pandemic's Devastation Continues. Available online at: https://www.hsph.harvard.edu/deans-office/2021/04/28/the-pandemics-devastation-continues/ (accessed October 1, 2021).

[B17] Honey F. M. Wong P. T. P. (2021). Hold on [Song]. Songs by Francine.

[B18] Hwang T. J. Rabheru K. Peisah C. Reichman W. Ikeda M. (2020). Loneliness and social isolation during the COVID-19 pandemic. Int Psychogeriatr. 32, 1217–1220. 10.1017/S1041610220000988 32450943 PMC7306546

[B19] International Network on Personal Meaning. (2019, May 2). Giving positive psychology away as a grassroots movement to combat the mental health crisis of depression and suicide. EinNews. Available online at: https://www.einnews.com/pr_news/484071351/giving-positive-psychology-away-as-a-grassroots-movement-to-combat-the-mental-health-crisis-of-depression-and-suicide (accessed October 1, 2021).

[B20] James W. (1902/1912). The Varieties of Religious Experience. Oxford, UK: Oxford University Press.

[B21] Jung C. G. (1949). Modern Man in Search of a Soul. London: K. Paul, Trench, Trubner and Co.

[B22] Kaufman S. B. (2020). Transcend: The New Science of Self-Actualization. New York, NY: Tarcher Perigee.

[B23] Khan S. Line K. Browne A. Reimer-Kirkham S. (eds.). (2021). Global Suffering and Uncertainty in the COVID-19 Pandemic: Exposing the fault Lines Through Narrative/Discourse Analysis. Frontiers. Available online at: https://www.frontiersin.org/research-topics/20758/global-suffering-and-uncertainty-in-the-covid-19-pandemic-exposing-the-fault-lines-through-narrative (accessed October 1, 2021).

[B24] Lee M. T. (2021). The ‘Flavorful Life' is bitter and sweet: pathways to flourishing in a time of suffering, in Global Wellbeing During the Pandemic: Honouring Ed Diener. International Network on Personal Meaning 11th Biennial International Meaning Conference (Toronto, ON).

[B25] Liu R. T. (2015). A developmentally informed perspective on the relation between stress and psychopathology: when the problem with stress is that there is not enough. J Abnorm Psychol. 124, 80–92. 10.1037/abn0000043 25688435 PMC4332562

[B26] Lomas T. Ivtzan I. (2016). Second wave positive psychology: exploring the positive-negative dialectics of wellbeing. J Happiness Stud. 17, 1753–1768. 10.1007/s10902-015-9668-y

[B27] Lukas E. (1990). Overcoming the “tragic triad.” Int. Forum Logother. 13, 89–96.

[B28] Marano H. E. (2021). The great reset. Psychology Today. Available online at: https://www.psychologytoday.com/ca/articles/202111/the-great-reset (accessed October 1, 2021).

[B29] Mayer C.-H. (2017). The Life and Creative Works of Paulo Coelho. New York, NY: Springer.

[B30] Mayer C.-H. (2021a). Angela Merkel's faith: its implications for political leadership and coping with Covid-19, in Presentation at the 11th International Virtual Meaning Conference, 6-8 August 2021 (Toronto, ON).

[B31] Mayer C.-H. (2021b). Leading with faith: Angela Merkel in psychobiographical perspective, in Reimagining Faith and Management: The Impact of Faith in the Workplace, eds. Po E. Klipatrick R. Pratt T. (Oxfordshire: Routledge), 32–46.

[B32] Mayer C.-H. Fouché P. J. P. (2021). Lessons learnt from Baruch Spinoza: shame and faith development in the light of challenges in contemporary society, in Shame 4.0. Investigating an Emotion in Digital Worlds and the Fourth Industrial Revolution, eds. Mayer C.-H. Vanderheiden E. Wong P. T. P. (Cham: Springer), 247–274.

[B33] Mayer C.-H. Vanderheiden E. Wong P. T. P. (2021). Shame 4.0. Investigating an Emotion in Digital Worlds and the Fourth Industrial Revolution. Cham: Springer.

[B34] Mayer C.-H. Viviers R. (2018). “Can one put faith and work in the same sentence?” Faith development and vocation of a female leader in the engineering profession. J Relig Health 57, 821–835. 10.1007/s10943-017-0404-2 28500501

[B35] McAdams D. P. (2013). The Redemptive Self: Stories Americans Live By. Oxford, UK: Oxford University Press.

[B36] Nield D. (2016). Here's why listening to sad music makes you feel better. Sciencealert. Available online at: https://www.sciencealert.com/new-research-reveals-the-pain-and-pleasure-of-listening-to-sad-music (accessed October 1, 2021).

[B37] Paulus N. (n.d.). How Listening to Sad Music Can Make You Feel Better. Musicto. Available online at: https://www.musicto.com/news/how-listening-to-sad-music-can-make-you-feel-better/ (accessed October 1, 2021).

[B38] Peek S. Casarella D. (2021). 15 business ideas to launch during a pandemic. USChamber.com. Available online at: https://www.uschamber.com/co/start/business-ideas/pandemic-business-ideas (accessed October 1, 2021).

[B39] Schlett J. (2021). Frontier Struggles: Rollo May and the Little Band of Psychologists Who Saved Humanism. The University of Akron Press.

[B40] Seligman M. E. P. (1999). The APA 1998 annual report. Am Psychol. 54:537–68.

[B41] Valinsky J. (2020). Business is booming for these companies during the COVID-19 pandemic. CTV News. Available online at: https://www.ctvnews.ca/health/coronavirus/business-is-booming-for-these-companies-during-the-covid-19-pandemic-1.4933907 (accessed October 1, 2021).

[B42] Vozza S. (2020). These 5 Myths About Resilience Might Be Hurting Your Ability to Cope. Fast Company. Available online at: https://www.fastcompany.com/90547087/these-5-myths-about-resilience-might-be-hurting-your-ability-to-cope (accessed October 1, 2021).

[B43] Weir K. (2020). Life after COVID-19: Making Space for Growth. American Psychological Association. Available online at: https://www.apa.org/monitor/2020/06/covid-life-after (accessed October 1, 2021).

[B44] Williams L. Rollins L. Young D. Fleming L. Grealy M. Janssen X.. (2021). What have we learned about positive changes experienced during COVID-19 lockdown? Evidence of the social patterning of change. PLoS One 16:e0244873. 10.1371/journal.pone.0244873 33400700 PMC7785245

[B45] Wong P. T. P. (1989). Personal meaning and successful aging. Can. Psychol. 30:516–25. 10.1037/h0079829

[B46] Wong P. T. P. (1993). Effective management of life stress: the Resource-Congruence Model, stress Medicine 9, 51–60. 10.1002/smi.246009011025855820

[B47] Wong P. T. P. (1995). A stage model of coping with frustrative stress, in Biological Perspectives on Motivated Activities, ed. Wong R. (New York, NY: Ablex Publishing), 339–378.

[B48] Wong P. T. P. (2009a). Existential positive psychology, in Encyclopedia of Positive Psychology, Vol. 1, ed. Lopez S. J. (Hoboken, NJ: Wiley Blackwell), 361–368.

[B49] Wong P. T. P. (2009b). Viktor Frankl: Prophet of hope for the 21st century, in Existential Psychotherapy of Meaning: Handbook of Logotherapy and Existential Analysis, eds. Batthyany A. Levinson J. (Phoenix, AZ: Tucker and Theisen).

[B50] Wong P. T. P. (2011). Positive psychology 2.0: towards a balanced interactive model of the good life. Can. Psychol. 52, 69–81. 10.1037/a0022511

[B51] Wong P. T. P. (ed.). (2012). Toward a dual-systems model of what makes life worth living, in The Human Quest for Meaning: Theories, Research, and Applications, 2nd Edn (Oxfordshire: Routledge), 3–22.

[B52] Wong P. T. P. (2016). Self-transcendence: a paradoxical way to become your best. Int. J. Existen. Posit. Psychol. 6:9. Available online at: https://www.meaning.ca/ijepp-article/vol6-no1/self-transcendence-a-paradoxical-way-to-become-your-best/

[B53] Wong P. T. P. (2017a). Meaning-centered approach to research and therapy, second wave positive psychology, and the future of humanistic psychology. Human. Psychol. 45, 207–216. 10.1037/hum0000062

[B54] Wong P. T. P. (2017b). Coping and stress, in The SAGE Encyclopedia of Abnormal and Clinical Psychology, ed. Wenzel A. (New York, NY: Sage), 886–890.

[B55] Wong P. T. P. (2017c). Courage, faith, meaning, and mature happiness in dangerous times. Positive Living Newsletter. Available online at: http://www.drpaulwong.com/inpm-presidents-report-may-2017/ (accessed October 1, 2021).

[B56] Wong P. T. P. (2019a). Why and How I Developed the Positive Psychology of Suffering. Available online at: http://www.drpaulwong.com/why-and-how-i-developed-the-positive-psychology-of-suffering/ (accessed October 1, 2021).

[B57] Wong P. T. P. (2019b). What is the greatest need today? Responsibility is the key to surviving and thriving in dangerous time. Positive Living Newsletter. Available online at: http://www.drpaulwong.com/what-is-the-greatest-need-today-responsibility-is-the-key-to-surviving-and-thriving-in-dangerous-times/ (accessed October 1, 2021).

[B58] Wong P. T. P. (2019c). Foreword: from shame to wholeness: an existential positive psychology perspective, in The Bright Side of Shame: Transforming and Growing Through Practical Applications in Cultural Contexts, eds. Mayer C.-H. Vanderheiden E. (Cham: Springer), 5–9.

[B59] Wong P. T. P. (2020a). Made for Resilience and Happiness: Effective Coping With COVID-19 According to Viktor E. Frankl Paul Wong T. P.. Toronto, ON: INPM Press.

[B60] Wong P. T. P. (2020b). The maturing of positive psychology and the emerging PP2.0. Int. J. WellBeing 10:107–117. 10.5502/ijw.v10i1.8858486778

[B61] Wong P. T. P. (2020c). Existential positive psychology and integrative meaning therapy. Int. Rev. Psychiatry. 32:565–578. 10.1080/09540261.2020.1814703 33016788

[B62] Wong P. T. P. (2021a). Existential Positive Psychology (PP2.0) and global wellbeing: why it is necessary during the age of COVID-19. Int. J. Existent. Posit. Psychol. 10, 1–16. Available online at: https://www.meaning.ca/ijepp-article/vol10-no1/what-is-existential-positive-psychology-pp-2-0-why-is-it-necessary-for-mental-health-during-the-pandemic/

[B63] Wong P. T. P. (2021b). Meaning Conference 2021 summit symposium on mental health – introduction, in Mental Health. International Network on Personal Meaning 11th Biennial International Meaning Conference, ed. Wong P. T. P. (Toronto, ON).

[B64] Wong P. T. P. (2021c). Preface: Frankl's cure for a soulless psychology and a sick society, in Psychobiography of Viktor Frankl, eds. Krasovska N. Mayer C.-H. (Cham: Springer Publishing), 1–8.

[B65] Wong P. T. P. (2021d). The Frankl cure for the 21st century: Why self-transcendence is the key to mental health and flourishing. PsyArXiv [PsyArXiv Preprints]. 10.31234/osf.io/tbx3f

[B66] Wong P. T. P. (2021e). Foreword, in Positive Psychology Across The Life Span An Existential Perspective, ed. Worth P. (Oxfordshire: Routledge).

[B67] Wong P. T. P. Bowers V. (2018). Mature happiness and global wellbeing in difficult times, in Scientific Concepts Behind Happiness, Kindness, and Empathy in Contemporary Society, ed. Silton N. R. (Hershey, PA: IGI Global), 112–134.

[B68] Wong P. T. P. McDonald M. (2002). Tragic optimism and personal meaning in counselling victims of abuse. Pastor. Sci. 20, 231–249.

[B69] Wong P. T. P. Reker G. T. Peacock E. J. (2006a). A resource-congruence model of coping and the development of the coping schema inventory, in Handbook of Multicultural Perspectives on Stress and Coping, eds. Wong P. T. P. Wong L. C. J. (New York, NY: Spring Publications), 223–283.

[B70] Wong P. T. P. Tomer A. (2011). Beyond terror and denial: the positive psychology of death acceptance. Death Stud. 35, 99–106. 10.1080/07481187.2011.535377 24501830

[B71] Wong P. T. P. Wong L. C. J. Scott C. (2006b). Beyond stress and coping: the positive psychology of transformation, in Handbook of Multicultural Perspectives on Stress and Coping, eds. Wong P. T. P. Wong L. C. J. (New York, NY: Spring Publications), 1–26.

[B72] Wong P. T. P. Worth P. (2017). The deep-and-wide hypothesis in giftedness and creativity. Psychol. Educ. 54, 11–23. Available online at: http://psychologyandeducation.net/pae/index.php/pae/Vol_54_No_3_4_20179104650

[B73] Wong P. T. P. Yu T. T. F. (2021). Existential suffering in palliative care: an existential positive psychology perspective. Medicina 57:924. 10.3390/medicina57090924 34577847 PMC8471755

[B74] Worth P. (ed.). (2021). Positive Psychology Across the Life Span: An Existential Perspective. Oxfordshire: Routledge.

